# Reconstitution Properties of Thymus Stem Cells in Murine Fetal Liver

**DOI:** 10.1155/1994/71860

**Published:** 1994

**Authors:** Hilary J. Mckenna, Nicholas M. Birchall, James D. Watson

**Affiliations:** Department of Molecular Medicine School of Medicine University of Auckland Auckland New Zealand

**Keywords:** Thymus, stem cells, fetal liver, cytokines

## Abstract

Injection of day-12 murine fetal liver cells into thymus lobes of Thy-1 congenic adult recipients results in a wave of thymocyte development. The kinetics of repopulation by donor cells reaches a peak after 20–25 days. The frequency of thymic stem cells (TSC) in day-12 fetal liver was estimated, by limit dilution, as 1 in 4x10^4^ cells. Within 8 hr of injection into a thymus lobe, fetal liver TSC commit to T-cell development, losing stem-cell activity. When fetal liver cells are maintained in culture for 7 days, with no exogenous cytokines added, and then injected intra-thymically (I.T.), thymus recolonization is not observed. However, TSC can be maintained in culture for 7 days with IL-1*β*, IL-3, IL-6, or LIF added, alone or in combination, with steel factor (SLF). Poisson analysis of fetal liver cells cultured with SLF and IL-3 together revealed a precursor frequency of 1 in 1.8x 10^5^ cells. In contrast, the frequency of TSC in adult bone marrow was estimated by limit dilution as 1 in 12,000 cells.


*Developmental Immunology*, 1994, Vol 3, pp. 247–256
Reprints available directly from the publisher
Photocopying permitted by license only

© 1994 Harwood Academic Publishers GmbH
Printed in Singapore

### Reconstitution Properties of Thymus Stem Cells in Murine Fetal Liver

HILARY J. McKENNA, NICHOLAS M. BIRCHALL and JAMES D. WATSON*

*Department of Molecular Medicine, School of Medicine, University of Auckland, Auckland, New Zealand*

Injection of day-12 murine fetal liver cells into thymus lobes of Thy-1 congenic adult recipients results in a wave of thymocyte development. The kinetics of repopulation by donor cells reaches a peak after 20–25 days. The frequency of thymic stem cells (TSC) in day-12 fetal liver was estimated, by limit dilution, as 1 in $4 \times 10^4$ cells. Within 8 hr of injection into a thymus lobe, fetal liver TSC commit to T-cell development, losing stem-cell activity. When fetal liver cells are maintained in culture for 7 days, with no exogenous cytokines added, and then injected intra-thymically (I.T.), thymus recolonization is not observed. However, TSC can be maintained in culture for 7 days with IL-1$\beta$, IL-3, IL-6, or LIF added, alone or in combination, with *steel* factor (SLF). Poisson analysis of fetal liver cells cultured with SLF and IL-3 together revealed a precursor frequency of 1 in $1.8 \times 10^5$ cells. In contrast, the frequency of TSC in adult bone marrow was estimated by limit dilution as 1 in 12,000 cells.

KEYWORDS: Thymus, stem cells, fetal liver, cytokines.

### INTRODUCTION

Thymus stem cells (TSC) are functionally defined here as those cells that have the capacity to seed the thymus and differentiate to functional thymus-derived (T) lymphocytes. Hematopoietic stem cells isolated from mouse fetal liver or bone marrow have been reported to express low levels of Thy-1 antigen (Thy-$1^{low}$), the Sca-1 antigen, but not the lineage markers Gr-1, Mac-1, B220, CD4, and CD8 ($Lin^-$), and it has been estimated that these cells represent 0.05–0.1% of the total population (Spangrude et al., 1988a; 1988b; Ikuta et al., 1990). Thy-$1^{low}$ Sca-$1^+$ $lin^-$ cells have the capacity to form day-12 spleen colonies (CFU-S) when injected intravenously into lethally irradiated adult mice, and to differentiate into T lymphocytes when injected into adult thymic lobes (Spangrude et al., 1988b). The earliest thymocyte progenitor population found in adult thymus appears to express low levels of the CD4 antigen (Wu et al., 1991).

A number of growth factors including *Steel* factor (SLF) (Huang et al., 1990; Williams et al., 1990; Zsebo et al., 1990), leukemia inhibitory factor (LIF) (Williams et al., 1988; Fletcher et al., 1990; 1991), interleukin-3 and -6 (Bodine et al., 1989; Chervenak and Altazan, 1990), and interleukin-7 (Namen et al., 1988; Watson et al., 1989) have been reported as having an effect on stem cells or early progenitor cell survival. Many of these are produced by stromal cells of the bone marrow. The production of cytokines by the stromal cells of fetal liver that may influence haemotopoietic stem cells has not been well documented. SLF exists in a membrane anchored and soluble form, is the ligand for the ci-*kit* proto-oncogene, and is the gene product of the murine *Steel* locus on chromosome 10 (Huang et al., 1990; Williams et al., 1990; Zsebo et al., 1990). The hematologic phenotype of mice with mutations of the *Steel* locus is characterized by a reduced number of hematopoietic stem cells, macrocytic anaemia, and a reduction of tissue mast cells (Russell, 1979). A normal number of both T and B lymphocytes is found in the peripheral lymphoid organs of *Steel* mice (Russell, 1979). SLF synergizes with IL-7 to enhance the growth of pre-B colonies in semisolid cultures of murine bone marrow (McNiece et al., 1991). SLF by itself stimulates the proliferation of bone marrow-derived murine spleen colony-forming cells (CFU-S) *in vitro* and shows synergy in combination with IL-1 and IL-3 (de Vries et al.,

*Corresponding author.

1991). Most *in vitro* clonogenic cells of the bone marrow as well as CFU-S express c-*kit*. The exception are the *in vivo* colony-forming cells responsive to IL-7 (Ogawa et al., 1991). A population of primitive stem cells that do not express c-*kit*, but give rise to c-$kit^+$ cells *in vitro* have been described (Kodama et al., 1992). Anti c-*kit* antibody treatment severely depleted bone marrow cultures of the cells that give rise to day-12 CFU-S. The cultures recovered to pretreatment CFU-S levels on return to normal growth conditions.

We show here that cells from day-12 unfractionated murine fetal liver repopulate the thymic lobes of irradiated adult mice with distinctive kinetics expected of TSC. A limiting dilution analysis of this colonization event has been performed. Using adoptive transfer to secondary recipients, we have studied the loss of stem-cell activity of these cells upon entry to the thymic environment. We conclude that commitment to the T-cell lineage is rapid, occuring within a few hours, and as a result of this, the ability to transfer stem-cell activity is lost. TSC in day-12 fetal liver can be maintained *in vitro* for 7 days provided the culture is supplemented with IL-1$\beta$, IL-3, IL-6, or LIF in both the presence and absence of SLF. A limit dilution analysis of TSC in fetal liver after 1 week in culture with SLF and IL-3 revealed this cytokine combination had not induced a net increase in the number of TSC.

### RESULTS

### Kinetics of Thymus Lobe Reconstitution

The following experiments establish the criteria we use to characterize TSC and are similar to studies reported elsewhere (Goldschneider et al., 1986; Scollay et al., 1986; Spangrude et al., 1988b). The effect of whole-body sublethal irradiation (750 rads) on adult thymocyte numbers was examined and a loss of more than 95% of thymocytes occurred over 3 days from each thymic lobe. After 5 days, thymocyte numbers began to increase and reached a plateau level of approximately $2\text{–}4 \times 10^7$ cells per lobe on day 12. This effect has been reported by a number of people (Takada et al., 1969; reviewed Anderson and Warner, 1976; Hirokawa et al., 1985; Mulder et al., 1985) and was observed whether or not the thymus had been intra-thymically (I.T.) injected with donor cells.

To follow the kinetics of the recolonization process using the expression of the Thy-1 marker, a single-cell suspension was prepared from day-12 embryonic liver of C57BL/Thy1.2 mice. Groups of 6–8-week-old C57BL/Thy1.1 mice were irradiated (750 rads) and after 3 hours, $5 \times 10^5$ cells were injected into one thymus lobe of each recipient. The second thymus lobe served as a control. Colonization of this lobe by donor cells was rarely seen, confirming observations that TSC show very limited migration from the lobe they are injected into (Goldschneider et at., 1986; Scollay et al., 1986). Beginning at day 8 after transfer, and continuing until day 30, C57BL/Thy1.1 recipients were sacrificed, and control and injected lobes separately removed and analyzed for the presence of $Thy1.1^+$ and $Thy1.2^+$ cells. The data presented in Fig. 1 are a summary of the percentage of both $Thy1.1^+$ and $Thy1.2^+$ cells found in the injected lobes of individual recipients. From day 10 and continuing to around day 22, there occurred a steady increase in $Thy1.2^+$ cells, with a concomitant loss of $Thy1.1^+$ cells, until they consistently comprised greater than 90% of the $Thy1^+$ cells. At this point, mature donor-derived T cells were readily detectable in both spleen and lymph nodes (data not presented). This pattern of reconstitution was similar for fetal

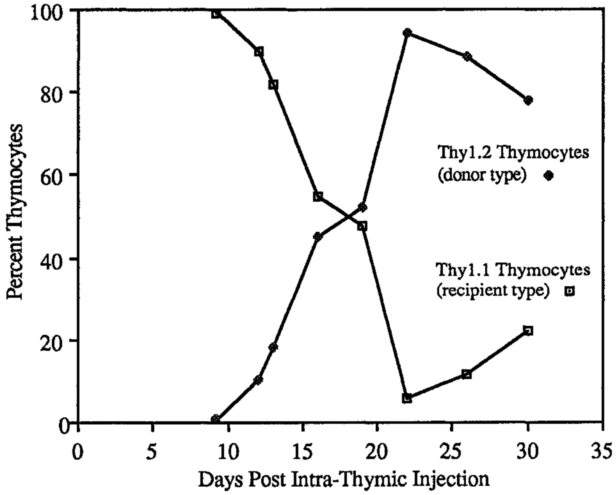

FIGURE 1. The kinetics of appearance of donor-derived Thy-$1^+$ cells after intra-thymic transfer of fetal liver cells. $5 \times 10^5$ fetal liver cells prepared from C57BL/Thy1.2 mice were injected intra-thymically into one thymic lobe of sublethally irradiated adult C57BL/Thyl.1 mice. On designated days, the mice were sacrificed, both thymic lobes were removed, and the percentage of $Thy1.1^+$ cells and $Thy1.2^+$ cells determined. The data represent an example of three experiments performed using cells from day-12 embryos.

liver cells from day-11 and -13 embryos (data not presented).

### Effect of Cell Numbers on Thymus Lobe Reconstitution

Groups of adult C57BL/Thy1.1 mice were irradiated (750 rads) and 3 hours later injected I.T. in one thymus lobe with a limiting number of day-12 fetal liver cells. The number of donor cells ranged from $3.13 \times 10^3$ per lobe. Twenty-one days after injection, thymus lobes were harvested and the proportion of Thy1.1$^+$ and Thy1.2$^+$ cells determined. A wide range in the proportion of donor-derived cells was seen in each group, and this was independent of the number of cells injected. The lowest level observed was just 3% repopulation in a mouse in the group that received $2.5 \times 10^4$ cells. Any lobe that contained a minimum of 1% donor cells was scored positive for colonization. The findings have been presented in Figs. 2(A) and 2(B). When subjected to a Poisson analysis, the data followed single-hit kinetics. For each point plotted, between 20 and 33 mice were analyzed. The interpretation of these results is that a single cell is capable of recolonizing a thymus lobe, and that the frequency of TSC in day-12 fetal liver is around 1 in $4 \times 10^4$ cells.

### Loss of Reconstitution Potential of Thymus Stem Cells

As stem cells differentiate in the thymus, they appear to lose their thymic recolonization potential (reviewed by Scollay et al., 1988). The kinetics by which stem cells lost their ability to colonize the thymus was analyzed. A group of C57BL/Thy1.1 adult mice was irradiated (750 rads) and 3 hours later injected I.T. in one thymus lobe with $1 \times 10^6$ liver cells from day-12 C57BL/Thy1.2 embryos. After 1, 8, 24, 48, and 96 hours, mice from this group were sacrificed, the injected lobes harvested, and single-cell suspensions prepared. A second group of adult C57BL/Thy1.1 mice was irradiated (750 rads) and one thymus lobe of each injected with up to $5 \times 10^6$ cells from the cell suspensions. Although it was impossible to quantitate accurately the number of donor-derived cells in each group, we estimated that the $5 \times 10^6$ cells would contain sufficient numbers of fetal liver cells or their progeny to reconstitute an adult thymus lobe if the cells retained their original reconstitution ability. The results of a typical experiment are presented in Fig. 3. At days 12, 15, and 20, Thy1.2$^+$ cells were detected in thymus lobes injected directly with $1 \times 10^6$ fresh day-12 fetal liver cells or with $5 \times 10^6$ thymocytes prepared 1 hour after the first I.T. injection. This also served as a control, demonstrating that fetal liver cells diluted by injection into an adult thymus lobe could be detected by secondary transfer. No significant numbers of Thy1.2$^+$ cells were detected in thymus lobes when donor cells prepared 8, 24, or 48 hr after the first I.T. injection were transferred into secondary recipients.

### Survival of Thymic Stem Cells in Culture

Numbers of cytokines were examined for their ability to support fetal liver TSC in culture. When day-12 fetal liver cells were cultured in medium supplemented separately with 20 ng/ml of IL-1$\beta$, IL-3, IL-6, IL-7, or LIF, minimal proliferation was observed (as with cultures with no cytokines added). SLF, however, induced significant proliferation over 7 days, resulting in cell numbers approximately five-fold above those obtained with medium alone. This SLF-induced increase was further augmented with the addition of any of the five cytokines (Fig. 4). Interestingly, morphological differences were evident in these cultures. In cultures containing no cytokines along with those supplemented with IL-1$\beta$, IL-3, IL-6, IL-7, or LIF, monolayers of adherent stromal type cells grew with clusters of closely attached, rounded, non adherent cells. In cultures containing SLF (either alone or in conjunction with another cytokine), the outgrowth of the adherent stromal layer was markedly inhibited. When cells were harvested 5 to 7 days after culture without cytokines added, and injected I.T., repopulation as reflected by a wave of appearance of donor Thy1.2$^+$ thymocytes was not observed. Up to $3–5 \times 10^5$ viable cells recovered from cell cultures were routinely injected in these experiments. SLF and IL-7 alone or in conjunction with SLF also failed to maintain the TSC in culture for 7 days. However, when $3 \times 10^5$ cells from 7-day cultures supplemented with IL-1$\beta$, IL-3, IL-6, or LIF, alone or in addition to SLF, were injected I.T., donor-derived thymocytes were detected 21 days after injection, suggesting that these conditions had all acted to maintain stem-cell viability (data not shown). Fetal liver cells grown in a combination of SLF and IL-3 were subjected to a limit dilution assay to determine whether these culture conditions had induced TSC proliferation. At day 21, the mice were sacrificed

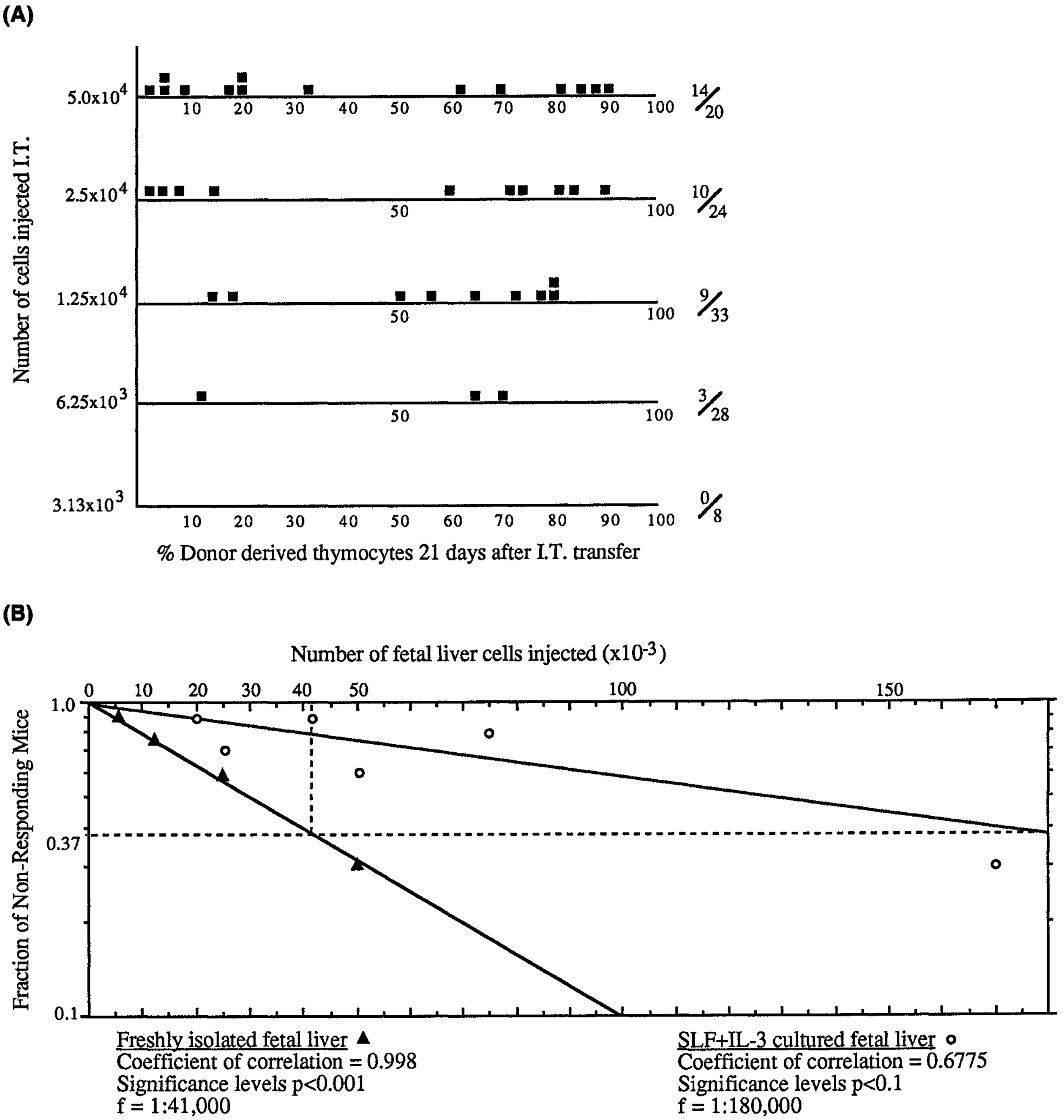

FIGURE 2. (A) The effect of limiting cell numbers on thymic lobe reconstitution. Sublethally irradiated C57BL/Thy1.1 adult mice were intra-thymically injected with limiting numbers of day-12 C57BL/Thy1.2 fetal liver cells, ranging from $5 \times 10^4$ down to $3.13 \times 10^3$ cells. Twenty-one days after injection the mice were sacrificed and the proportion of Thy1.1$^+$ and Thy1.2$^+$ cells was determined in the injected lobe. Any lobe showing greater than 1% Thy1.2$^+$ cells at day 21 was scored as showing repopulation. (The solid square represents one mouse, and the fractions to the right denote the number of mice showing repopulation relative to the number injected.) (B) Poisson analysis of the limit dilution data. The fraction of non-repopulated mice from Fig. 2(A) was plotted against the number of cells injected (solid triangles). Poisson analysis of limit dilution performed with SLF + IL-3 cultured fetal liver: Day- 12 fetal liver cells from C57BL/Thy1.2 embryos were isolated and placed in culture supplemented with SLF + IL-3 as for Fig. 4. After 7 days, the cells were harvested and the viable cells counted. The increase in net cell number was noted. Numbers ranging from $1.7 \times 10^5$ down to $2.08 \times 10^4$ were injected I.T. and the mice were sacrificed and analyzed on day 21. Fifty-seven mice were analyzed. The fraction of nonrepopulated mice were plotted against the number of cells injected (open circle). (The solid square is the frequency of TSC in adult bone marrow. Each group contained 20 mice.)

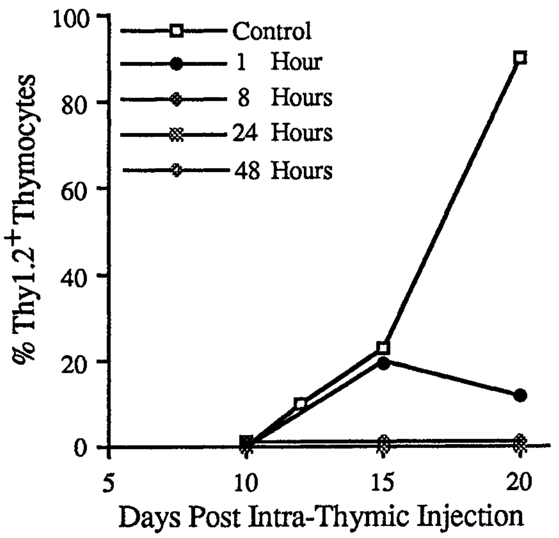

FIGURE 3. The loss of reconstitution potential of thymic stem cells. C57BL/Thy1.1 adult mice were sublethally irradiated and 3 hr later were injected I.T. with $1 \times 10^6$ day-12 C57BL/Thy1.2 fetal liver cells. At designated times (1, 8, 24, and 48 hr after this primary injection), the mice were sacrificed and the injected lobes were removed. A single-cell suspension was made and $5 \times 10^6$ viable thymocytes were transferred I.T. into secondary sublethally irradiated C57BL/Thy1.1 adults. The percent of Thy1.2$^+$ thymocytes was determined 12, 15, and 20 days after this secondary transfer. The control group received only the primary injection.

and the thymic lobes assayed; see Fig. 2(B). The frequency of TSC as determined by Poisson analysis had fallen to 1 in $1.8 \times 10^5$ cells. As there was a three- to fourfold increase in total cell numbers over the 7 days in culture, we conclude that the combination of SLF and IL-3 had maintained the viability of the TSC, but there had been no significant net increase in TSC number.

### Frequency of Thymus Stem cells in Adult Bone Marrow

To determine the frequency of TSC in adult bone marrow, groups of 20 adult C57BL/Thy1.1 mice were irradiated (750 rads) and injected with Thy 1.2 adult bone marrow cells. The number of donor cells ranged from $2 \times 10^3$ to $8 \times 10^4$ per lobe. Twenty-one days after injection, thymus lobes were harvested and the proportion of Thy1.1$^+$ and Thy1.2$^+$ cells determined. The results have also been included in Fig. 2(B). Unlike the results using fetal liver as donor cells, the lowest level of repopulation was 30% Thy1.2 cells in a mouse that had received $5 \times 10^3$ cells. When subjected to a Poisson analysis, the data followed single-hit kinetics with the frequency of TSC around 1 in 12,000 cells.

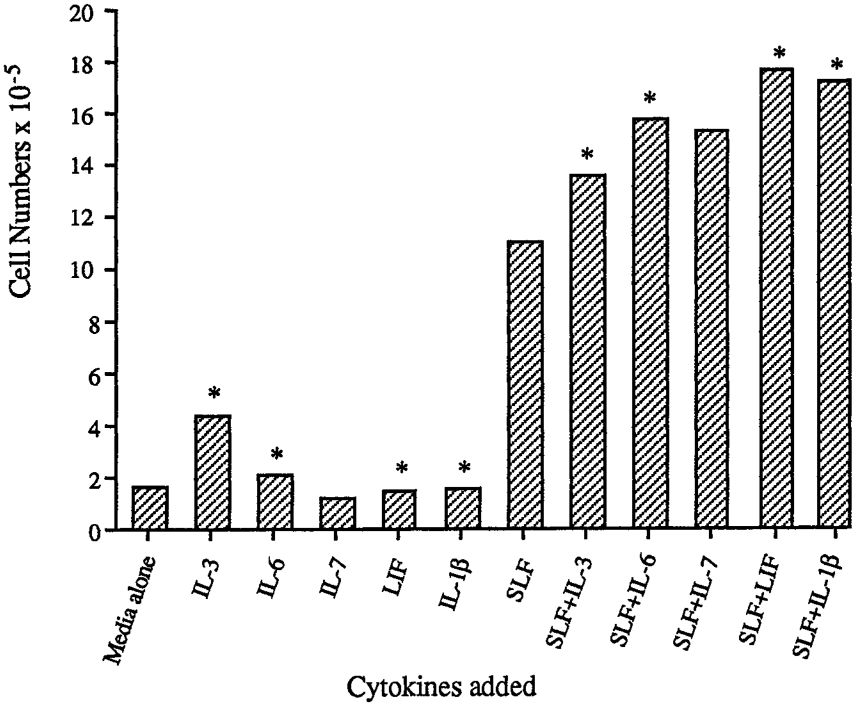

FIGURE 4. Proliferative response of day-12 fetal liver to cytokines and the detection of thymic reconstitution by cytokine grown cells. Day-12 C57BL/Thy1.2 fetal liver was removed and a single-cell suspension was prepared. Cells were plated in 24-well plates at $5 \times 10^5$ viable cells per milliliter. The designated cytokines were all added at 20–30 ng/ml. The medium was replaced 5 days after establishing the cultures, and on day 7, the cultures were harvested and the number of viable cells determined. Each figure plotted represents the mean of 7–10 wells. Sublethally irradiated C57BL/Thy1.1 adults (6–10 per group) were injected I.T. with $3\text{–}5 \times 10^5$ viable cells from each culture condition. The percent of donor-derived Thy1.2$^+$ cells was determined on day 21. The groups with mice showing repopulation are marked with an asterisk(*).

### DISCUSSION

The strategy used here to identify cells with thymic stem-cell activity has been the colonization of a sublethally irradiated adult thymus lobe following direct injection of donor cells (Goldschneider et al., 1986; Scollay et al., 1986; Spangrude et al., 1988b). Injection of $5 \times 10^5$ day-11 to -13 fetal liver cells from Thy1.2 donors into Thy1.1 recipients results in a lag period of some 8–10 days during which donor cells are virtually undetectable in thymus lobes (Fig. 1). The donor cells contain no Thy-$1^{high}$ cells (data not shown). A similar lag period has been observed after injection of adult bone marrow I.V. and I.T. (Boersma et al., 1981; Scollay et al., 1986; Goldschneider et al., 1986; Mulder et al., 1985), and fetal liver I.V. (Boersma, 1983). Between day 10 and 12, Thy$1.2^+$ cells begin to accumulate, and increase until day 20–25, when mature T cells begin to exit the thymus lobe. As these cells exit, a wave of thymocytes of host origin replace them. This kinetic pattern has been used by others to define stem cells with the property of thymic reconstitution (Goldschneider et al., 1986; Scollay et al., 1986; Spangrude et al., 1988b; Ikuta et al., 1990). The number of stem cells that seed the thymus under these conditions is likely to be small. After I.T. transfer of 200–7000 bone marrow–derived stem cells, Spangrude and Scollay (1990) noted an earlier appearance of donor Thy-$1^+$ cells that quickly reached a plateau by day 16–17. The delayed appearance of thymocytes expressing high levels of Thy-1 antigen appears to represent a period of proliferation. During ontogeny, the first thymic stem cells initiate colonization of the fetal thymus around day 11 of gestation, and mature into Thy-$1^+$ cells without the 10-day lag period seen in adult thymus colonization (Jotereau et al., 1987), suggesting that either fetal and adult thymic stem cells differ or the environments in to which they seed differ.

We have attempted to estimate the frequency of TSC in unfractionated fetal liver by limit dilution. Decreasing numbers of fetal liver cells were injected into thymic lobes of groups of irradiated adult recipients. When examined 21 days after injection, the proportion of donor-derived Thy-$1^+$ cells in each recipient varied widely; see Fig. 2(A). This effect appeared independent of the number of fetal liver cells initially injected. There are several possible explanations for this. Firstly, when a limited number of cells was injected, host-derived bone marrow stem cells that survived irradiation also seed the thymus, thus creating a chimeric lobe. Secondly, some form of competition may exist between host- and donor-derived TSC, not evident when a relatively large number of donor cells are injected (Fig. 1). Thirdly, TSC may be a heterogenous population. Some have the capacity to give rise to large numbers of progeny, whereas others yield a much smaller clone size. Finally, as more cells seed in the thymus, an environment for furthur seeding of TSC may be created that, in the case of limited availability of donor-type stem cells, would mean that host-derived TSC cells would be favored.

By scoring lobes that contained a minimum 1% donor-type cells as positive, single-hit kinetics were observed. Poisson analysis yielded an estimate of 1 in $4 \times 10^4$, or 0.0025% for TSC; see Fig. 2(B). This is lower than estimates of stem cells in adult bone marrow or day-14 fetal liver (Katsura et al., 1986; Spangrude et al., 1988b; Ikuta et al., 1990). As a result, we then analyzed TSC in adult bone marrow using limit dilution and observed a frequency of 1:12,000; see Fig. 2(B). Thus, there is a real difference in the frequencies of TSC in fetal liver and adult bone marrow. Boersma (1983) has demonstrated that the frequency of both prothymocytes and day-9 CFU-S in fetal liver is considerably less than that in bone marrow. This effect was reported after I.V. transfer and may reflect a poorer homing ability rather than a lower frequency and fetal liver TSC may not seed the adult thymus as efficiently as TSC in adult bone marrow. The difference between the estimates of 1 in 12,000 for TSC frequency in bone marrow and 1 in 41,000 for fetal liver might be explained purely by the different source of these TSC, the fact that these estimates were made on different days post–I.T. injection, or the level of detection of Thy-$1^+$ cells. The estimates of the frequency in day-14 fetal liver; 1 in 14,000 by Ikuta et al. (1900) and 1 in 6,000 (the number required to give 50% of the mice repopulated) by Katsura et al. (1986) are difficult to compare. It is unclear at what time point Ikuta et al. (1990) analyzed their mice. Katsura et al. (1986) analyzed their mice 21 days post–I.T. It has been demonstrated that early fetal liver is primarily involved in the production of cells of the erythroid lineage (Metcalf and Moore, 1971). Stem cells present in the liver, at or around day 12, may favor commitment to cells of the erythroid/myeloid lineage, thus limiting the proportion of stem cells available for the T-cell lineage.

It has been reported that colonization of a thymic lobe can result from the seeding of very few cells.

Wallis et al. (1975) demonstrated that an irradiated thymus could be entirely repopulated with few stem cells after intra-venous (I.V.) transfer of bone marrow cells. The same conclusion was reached by Ezine et al. (1984). In some mice they observed total lobe repopulation resulted from just one cell. Injection of $5 \times 10^4$ fetal liver cells resulted in 70% of the thymus lobes showing repopulation at day 21; see Fig. 2(A). This observation supports the observations of Wallis et al. (1975) and Ezine et al. (1984). Recently, de Vries et al. (1992) reported that thymic repopulation was observed in four out of nine mice that had been injected I.T. with just one c-*kit*$^+$ cell purified from adult bone marrow.

The loss of the reconstitution capacity of TSC was followed by a secondary adoptive transfer procedure (Fig. 3). The interesting observation was that after fetal liver cells had been in the thymus for 8 hr, their ability to recolonize another thymus lobe was lost. We argue from these data that following entry to the thymus, these cells rapidly undergo differentiation events that commit them to the T-cell lineage (even though Thy-1$^+$ cells cannot be detected for 10 days) with a concomitant loss of stem-cell activity. We have observed a similar phenomenon using adult bone marrow or c-*kit*$^+$ cells (de Vries et al., 1992) purified from adult bone marrow (unpublished data). In an adult thymus, TSC activity contained within the most recent immigrants, the CD4$^{low}$/CD8$^-$ cells and the double negatives (CD4$^-$/CD8$^-$) can be transferred to adult recipients (reviewed by Scollay et al., 1988; Wu et al., 1991). Thus, it is unclear as to why a secondary transfer reveals the rapid loss of TSC. It is difficult to accurately measure the number of donor cells transferred into the secondary recipient lobe. By injecting up to $10^6$ cells into the lobe of the primary recipient, and up to $5 \times 10^6$ cells into the lobe of the secondary recipient, by any reasonable calculation, we expect to be still transferring in excess of 100,000 donor cells after 96 hr. Using day-13–14 fetal thymocytes as a source of TSC, we have been unable to see adult lobe repopulation. Up to $5 \times 10^5$ day-13 and $1 \times 10^6$ day-14 fetal thymocytes were injected I.T. and recipient mice were examined between day 16 and day 21 after injection. This suggests a very rapid commitment by the fetal liver-derived stem cells within the fetal thymic lobes, although Guidos et al. (1989) have reported a wave of thymic repopulation after I.T. injection of $1–3 \times 10^5$ CD4$^-$/CD8$^-$ fetal thymocytes. It is possible that the inability to adoptively transfer TSC may reect pressure to reform the thymic environment damaged by irradiation. The possibility that stem-cell activity cannot be transferred because the cells have exited the thymus to traffic through the bone marrow, before reseeding in the thymus, can be discounted, as donor-derived Thy-1$^+$ cells are only rarely detected in the noninjected lobe. Sublethal irradiation of adult mice results in the loss of greater than 95% of thymocytes over 3 days and essentially collapses the steady-state microenvironments within the thymus (Takada et al., 1969; reviewed by Anderson and Warner, 1976). The fact that injection of normal adult thymocytes into a thymus of an irradiated recipient does not immediately restore the integrity of the thymus implies that there is a preciseness to thymic structure that depends on clonal development of recent stem-cell immigrants (Scollay et al., 1988). The rapid loss of reconstitution potential of TSC (Fig. 3) reinforces this. The I.T. injection of thymocytes already committed to a temporal sequence of events associated with maturation may be successful only if by chance cells lodge in an appropriate existing microenvironment or the thymocytes are immature enough to retain reconstitution properties.

SLF induced significant proliferation of fetal liver over 7 days in culture (Fig. 4), whereas IL-1$\beta$, IL-3, IL-6, IL-7, and LIF maintained the viability of only a fraction of the cells. The inability of SLF grown fetal liver cells to reconstitute the thymus suggests that either the TSC have died in this culture system or they have been induced to differentiate and commit to a certain lineage, thus losing their TSC activity. IL-1$\beta$, IL-3, IL-6, and LIF failed to produce significant proliferation over 7 days; however, these cytokines all established conditions that were favourable for the maintenance of viable TSC. These TSC may express receptors for all these cytokines (Dexter et al., 1990), or these factors may all act through an intermediate cell, resulting in the production of a further cytokine(s). The addition of SLF with any of these cytokines resulted in enhanced proliferation of the fetal liver cells. These cultures were also able to maintain the TSC in culture. Cultures supplemented with IL-7 or SLF, or the two in combination, failed to support the TSC. Growth of bone marrow cells with IL-7 alone or in combination with SLF may favor early B-cell development (Namen et al., 1988; McNiece et al., 1991). The combination of SLF and IL-3 has been shown to cause proliferation of stem cells purified from adult murine bone marrow as detected by a net increase in day-14 CFU-S (de Vries et al., 1991). However, this combination did not bring about a net increase in TSC in day-12 fetal liver, though it is quite possible that there had been some stem-cell self-renewal that was counteracted by some stem-cell death; see Fig. 2(B). This may reflect the influence of cytokines produced by the range of cells other than stem cells present in fetal liver. Efforts are underway now to purify the TSC cells in fetal liver to follow the effect SLF and IL-3 have on a purified population.

### MATERIALS AND METHODS

### Mice

C57BL/KATHY (Thy1.1) and C57BL/10J (Thy1.2) mice were bred at the School of Medicine, Auckland University, New Zealand. Mice were mated overnight and fetal liver isolated from day-11–13 embryos.

### Cell Suspensions

Freshly isolated fetal livers and adult thymuses were forced through a fine sieve to prepare single-cell suspensions. In each case, numbers of viable cells were estimated using trypan blue exclusion.

### Flow Cytometry

Flow cytometry was performed on a fluorescence-activated cell sorter (FACS 440, Becton Dickinson, Mountain View, CA) equipped with dual 2- and 5-watt argon lasers. Either laser was used at 200-mw output using the 488-nm line. For each treatment, $1\times10^4$ viable cells were analyzed. All data were collected and analyzed on a MicroVAX computer using Consort 40 (Becton Dickinson) software.

### Monoclonal Antibodies

Staining for detection of Thy1.1 antigen was performed using saturating amounts of anit-Thy1.1 antibody, clone 22.1.D1, for 30 min at 4°C, followed by a fluoresceinated anti-mouse IgM (Tago, Auckland, New Zealand). Staining for detection of Thy1.2 antigen was performed using anti-Thy1.2 antibody directly conjugated to FITC (Becton Dickenson). The media used for washing the cells comprised PBS with 2% FCS (Life Technologies, Auckland, New Zealand) and 0.01% sodium azide. Propidium iodide was included in the final wash at 10 mg/ml, and used to exclude dead cells.

### Intrathymic Injections

C57BL/Thy1.1 mice were used as recipients at 6–10 weeks of age. These mice were sublethally irradiated (750 rads) using a Cobalt-60 source. Three hours later, mice were anesthetized using a combination of Ketalar (Parke-Davis, Caringbah, N.S.W., Australia) at 188 $\mu$g/gm body weight and Rompun (Bayer, Auckland, N.Z.) at 6.4 $\mu$g/gm body weight, injected intramuscularly. An incision was made in the thoracic region, exposing the sternum, and the anterior third of the sternum was bisected. Cell suspensions were then injected using a Hamilton syringe (Reno, Nv), equipped with a 30-gauge needle (Smith and Nephew MPL, Chicago, Il), in 10-$\mu$l volumes into one thymic lobe. The incision was closed with Michel clips. All donor cells were derived from day-11–13 unfractionated fetal liver from C57BL/Thy1.2 mice. At designated times after transfer of the cells, the mice were sacrificed and thymus lobes harvested, single-cell suspension were prepared, and expression of the surface markers Thy1.1 and Thy1.2 was examined.

### Fetal Liver Cell Culture

Single-cell suspensions of C57BL/Thy1.2 day-12 fetal liver were prepared. For each culture $5\times10^5$ viable cells as determined by trypan blue exclusion were placed in 1 ml of DMEM containing 10% fetal calf serum, additional amino acids, and $5\times10^{-5}$ M 2-mercaptoethanol. The designated cytokines (Immunex, Seattle, WA) were addded at 20–30 ng/ml. After 5 days in culture, the medium was replaced. After 7 days, the cells were harvested from the wells and counted using trypan blue to determine cell viability.

### ACKNOWLEDGMENTS

We thank Roland Scollay (Walter and Eliza Hall Institute of Medical Research, Melbourne, Australia) for help with the intra-thymic assay system, Louise Moffatt for technical assistance, and John Marbrook for helpful discussion. We are indebted to Immunex (Seattle, WA) for supplying us with cytokines. This work was supported by the Health Research Council of New Zealand, and a fellowship to H.

McKenna from the Taranaki branch of the New Zealand Cancer Society.

*(Received March 8, 1993)*

*(Accepted August 5, 1993)*

### REFERENCES

Anderson R.E., and Warner N.L. (1976). Ionizing radiation and the immune response. Adv. Immunol. **24**: 215–335.

Bodine D.M., Karlsson S., and Nienhuis A.W. (1989). Combination of Interleukins 3 and 6 preserves stem cell function in culture and enhances retrovirus-mediated gene transfer into haematopoietic stem cells. Proc. Natl. Acad. Sci. USA **86**: 8897–8901.

Boersma W.J.A. (1983). Prothymocytes in mouse fetal liver. Thymus **5**: 419–428.

Boersma W.J.A., Betel I., Daculsi R., and Van der Westen G. (1981). Post- irradiation thymocyte regeneration after bone marrow transplantation. 1. Regeneration and quantification of thymocyte progenitor cells in the bone marrow. Cell Tissue Kinet. **14**: 179–196.

Chervenek R., and Altazan J.D. (1990). *In vitro* maintenance of bone marrow-resident T cell precursors in culture supplemented with exogenous IL-3. Cell. Immunol. **130**: 150–159.

de Vries P., Brasel K.A., Eisenman J.R., Alpert A.R., and Williams D.E. (1991). The effect of recombinant mast cell growth factor on purified murine hematopoietic stem cells. J. Exp. Med. **173**: 1205–1211.

de Vries P., Brasel K.A., McKenna H.J., Williams D.E., and Watson J.D. (1992). Thymus reconstitution by *c-kit* expressing hematopoietic stem cells purified from adult mouse bone marrow. J. Exp. Med. **176**: 1503–1509.

Dexter T.M., Heyworth C.M., Spooncer E., and Ponting I.L. (1990). The role of growth factors in self-renewal and differentiation of haemopoietic stem cells. Philos. Trans. Roy. Soc. London (Biol.) **327**: 85–98.

Ezine S., Weissman I.L., and Rouse R.V. (1984). Bone marrow cells give rise to distinct clones within the thymus. Nature **309**: 629–631.

Fletcher F.A., Moore K.A., Ashkenazi M., de Vries P., Overbeek P.A., Williams D.E., and Belmont J.W. (1991). Leukemia inhibitory factor improves survival of retroviral vector infected hematopoietic stem cells *in vitro*, allowing efficient long-term expression of vector-encoded human adenosine deaminase *in vivo*. J. Exp. Med. **174**: 837–845.

Fletcher F.A., Williams D.E., Maliszewski C., Anderson D., Rives M., and Belmont J.W. (1990). Murine leukemia inhibitory factor enhances retroviral-vector infection efficiency of haematopoietic progenitors. Blood **76**: 1098–1103.

Goldschneider I., Komschlies K.L., and Greiner D.L. (1986). Studies of thymocytopoiesis in rats and mice. I. Kinetics of appearance of thymocytes using a direct intrathymic adoptive transfer assay for thymocyte precursors. J. Exp. Med. **163**: 1–17.

Guidos C.J., Weissman I.L., and Adkins B. (1989). Developmental potential of $CD4^-8^-$ thymocytes. Peripheral progeny include mature $CD4^-8^-$ T cells bearing αβ T cell receptor. J. Immunol. **142**: 3773–3780.

Hirokawa K., Sado T., Kubo S., Kamisaku H., Hitomi K., and Utsuyama M. (1985). Intrathymic T cell differentiation in radiation bone marrow chimeras and its role in T cell emigration to the spleen. An immunohistochemical study. J. Immunol. **134**: 3615–3624.

Huang E., Nocka K., Beier D.R., Chu T., Buck J., Lahm H., Wellner D., Leder P., and Besmer P. (1990). The haematopoietic growth factor KL is encoded by the *Sl* locus and is the ligand for the *c-kit* receptor, the gene product of the W locus. Cell **63**: 225–233.

Ikuta K., Kina T., MacNeill I., Uchida N., Peault B., Chien Y., and Weissman I.L. (1990). A developmental switch in thymic lymphocyte maturation potential occurs at the level of hematopoietic stem cells. Cell **62**: 863–874.

Jotereau F., Heuze F., Salomon-Vie V., and Gascan H. (1987). Cell kinetics in the fetal mouse thymus: Precursor cell input, proliferation, and emigration. J. Immunol. **138**: 1026–1030.

Katsura Y., Kina T., Amagai T., Tsubata T., Hirayoshi K., Takaoki Y., Sado T., and Nishikawa S.I. (1986). Limiting dilution analysis of the stem cells for the T-cell lineage. J. Immunol. **137**: 2434–2439.

Kodama H., Nose M., Yamaguchi Y., Tsunoda J., Suda T., Nishikawa S., and Nishikawa S. (1992). In vitro proliferation of primitive hemopoietic stem cells supported by stromal cells: Evidence for the presence of a mechanism(s) other than that involving *c-kit* receptor and its ligand J. Exp. Med. **176**: 351–361.

McNiece I.K., Langley K.E., and Zsebo K.M. (1991). The role of recombinant stem cell factor in early B cell development. J. Immunol. **146**: 3785–3790.

Metcalf D., and Moore M.A.S. (1971). Embryonic aspects of haemopoiesis. In: Haemopoietic cells, Neuberger A. and Tatum E.L., Eds. (Amsterdam and London, North-Holland), pp. 195–210.

Mulder A.H., Visser J.W.M., and van den Engh G.J. (1985). Thymus regeneration by bone marrow cell suspensions differing in the potential to form early and late spleen colonies. Exp. Hematol. **13**: 768–775.

Namen A.E., Schmierer A.E., March C.J., Overell R.W., Park L.S., Urdal D.L., and Mochizuki, D.Y. (1988). B cell precursor growth-promoting activity. Purification and characterization of a growth factor active on lymphocyte precursors. J. Exp. Med. **167**: 988–1002.

Ogawa M., Matsuzaki Y., Nishikawa S., Hayashi S.I., Kunisada T., Sudo T., Kina T., Nakauchi H., and Nishikawa S.I. (1991). Expression and function of *c-kit* in hemopoietic progenitor cells. J. Exp. Med. **174**: 63–71.

Russell E.S. (1979). Hereditary anemias of the mouse: A review for geneticists. Adv. Genet. **20**: 357–459.

Scollay R., Smith J., and Stauffer V. (1986). Dynamics of early T cells: Prothymocyte migration and proliferation in the adult mouse thymus. Immunol. Rev. **91**: 129–157.

Scollay R., Wilson A., Di Amico A., Kelly K., Egerton M., Pearse M., Wu L., and Shortman K. (1988). Developmental status and reconstitution potential of subpopulations of murine thymocytes. Immunol. Rev. **104**: 81–120.

Spangrude G.J., Heimfeld S., and Weissman I.L. (1988a). Purification and characterization of mouse haematopoietic stem cells. Science **241**: 58–62.

Spangrude G.J., Müller-Sieburg C.E., Heimfeld S., and Weissman I.L. (1988b) Two rare populations of mouse $Thy\text{-}1^{lo}$ bone marrow cells repopulate the thymus. J. Exp. Med. **167**: 1671–1683.

Spangrude G.J., and Scollay R. (1990). Differentiation of hematopoietic stem cells in irradiated mouse thymic lobes. Kinetics and phenotype of progeny. J. Immunol. **145**: 3661–3668.

Takada A., Takada Y., Huang C.C., and Ambrus J.L. (1969). Biphasic pattern of thymus regeneration after whole-body irradiation. J. Exp. Med. **129**: 445–457.

Wallis V.J., Leuchars E., Chwalinski S., and Davies A.J.S. (1975). On the sparse seeding of bone marrow and thymus in radiation chimaeras. Transplantation **19**: 2–11.

Watson J.D., Morissey P.J., Namen A.E., Conlon P.J., and Widmer M.B. (1989). Effect of Interleukin 7 on growth of fetal thymocytes in culture. J. Immunol. **143**: 1215–1222.

Williams D.E., Eisenman J., Baird A., Rauch C., van Ness K., March C.J., Park L.S., Martin U., Mochizuki D.Y., Boswell H.S., Burgess G., Cosman D., and Lyman S.D. (1990). Identification of a ligand for the c-*kit* proto-oncogene. Cell **63**: 167–174.

Williams R.L., Hilton D.J., Pease S., Willson T.A., Stewart C.L., Gearing D.P., Wagner E.F., Metcalf D., Nicola N.A., and Gough N.M. (1988). Myeloid leukemia inhibitory factor maintains the developmental potential of embryonic stem cells. Nature **336**: 684–687.

Wu L., Scollay R., Egerton M., Pearse M., Spangrude G.J. and Shortman K. (1991). CD4 expressed on earliest T-lineage precursor cells in the adult murine thymus. Nature **349**: 71–74.

Zsebo K.M., Wypych J., McNeice I.K., Lu H.S., Smith K.A., Karkare S.B., Sachdev R.K., Yuschenkoff V.N., Birkett N.C., Williams L.R., Satyagal V.N., Tung W., Bosselman R.A., Mendiaz E.A., and Langley K.E. (1990). Identification, purification, and biological characterization of hematopoietic stem cell factor from buffalo rat liver-conditioned medium. Cell **63**: 195–201.

